# Potential causal role of synovial complement system activation in the development of post-traumatic osteoarthritis after anterior cruciate ligament injury or meniscus tear

**DOI:** 10.3389/fimmu.2023.1146563

**Published:** 2023-05-03

**Authors:** V. Michael Holers, Rachel M. Frank, Andrew Clauw, Jennifer Seifert, Michael Zuscik, Sakthi Asokan, Christopher Striebich, Michael R. Clay, Larry W. Moreland, Nirmal K. Banda

**Affiliations:** ^1^Division of Rheumatology, School of Medicine, University of Colorado Anschutz Medical Campus, Aurora, CO, United States; ^2^Department of Orthopedics and the Colorado Program for Musculoskeletal Research, School of Medicine, University of Colorado Anschutz Medical Campus, Aurora, CO, United States; ^3^Department of Pathology, School of Medicine, University of Colorado Anschutz Medical Campus, Aurora, CO, United States

**Keywords:** complement system, anterior cruciate ligament, medial meniscus tear, lateral meniscus tear, osteoarthiritis, synovium

## Abstract

Anterior cruciate ligament (ACL) injury and meniscal tear (MT) are major causal factors for developing post-traumatic osteoarthritis (PTOA), but the biological mechanism(s) are uncertain. After these structural damages, the synovium could be affected by complement activation that normally occurs in response to tissue injury. We explored the presence of complement proteins, activation products, and immune cells, in discarded surgical synovial tissue (DSST) collected during arthroscopic ACL reconstructive surgery, MT-related meniscectomy and from patients with OA. Multiplexed immunohistochemistry (MIHC) was used to determine the presence of complement proteins, receptors and immune cells from ACL, MT, OA synovial tissue vs. uninjured controls. Examination of synovium from uninjured control tissues did not reveal the presence of complement or immune cells. However, DSST from patients undergoing ACL and MT repair demonstrated increases in both features. In ACL DSST, a significantly higher percentage of C4d+, CFH+, CFHR4+ and C5b-9+ synovial cells were present compared with MT DSST, but no major differences were seen between ACL and OA DSST. Increased cells expressing C3aR1 and C5aR1, and a significant increase in mast cells and macrophages, were found in ACL as compared to MT synovium. Conversely, the percentage of monocytes was increased in the MT synovium. Our data demonstrate that complement is activated in the synovium and is associated with immune cell infiltration, with a more pronounced effect following ACL as compared to MT injury. Complement activation, associated with an increase in mast cells and macrophages after ACL injury and/or MT, may contribute to the development of PTOA.

## 1 Introduction

Osteoarthritis (OA) is the most common form of arthritis, impacting the quality of life for millions of people worldwide and constituting a major challenge for health systems. The etiology of OA is multifactorial. One of the most important modifiable risk factors in individuals that leads to early onset OA is the occurrence of a joint injury during participation in sports such as football and soccer (1), where tearing or spraining of the anterior cruciate ligament (ACL) and meniscal tear (MT), i.e., medial or lateral meniscal tear (MMT or LMT), are relatively common injuries. ACL tears represent more than 50% of knee injuries and affect more than 200,000 people in the United States each year, with direct and indirect costs greater than $7 billion annually (2, 3). Females are at three times higher risk of undergoing an ACL injury than males (4). In contrast, males experience two times as many meniscal injuries as females (5). The incidence of post-traumatic OA (PTOA) following ACL tear is as high as 87% (6, 7). Most patients with ACL tears are younger than 30 at the time of their injury, and these ACL injuries lead to early onset OA (8).

The mechanism of cartilage deterioration following ACL injuries and MT after surgery is not completely understood, but it is known that the articular cartilage sustains a considerable mechanical impact at the time of injury (9) and is exposed to an altered mechanical environment persistently after injury in both cases (10, 11). The medial meniscus is physically linked to the cartilage, and a torn meniscus is also one of the most common sports-related knee injuries. Although the exact incidence of MT is unknown, some estimates suggest that it is 60 per 100,000. Approximately 75% of symptomatic OA patients have prior meniscus injuries (12, 13). MT repair surgery is also one of the most common procedures performed after injury in the United States (14).

In addition to cartilage damage, the synovium can be affected by inflammatory processes after ACL injury or MT that is associated with chronic innate immune system activation, including that of the complement system (CS). This process could result in chronic changes in the synovium of the types normally associated with the development of an inflammatory arthritis such as rheumatoid arthritis (RA) (15). Complement is a potent effector arm of innate immunity. It is organized into three activation pathways including the classical pathway (CP), the lectin pathway (LP) and the alternative pathway (AP), the latter of which forms spontaneously on self surfaces and other targets that lack sufficient levels of regulatory/inhibitory proteins. All three complement activation pathways converge on the proteolytic activation of complement component 3 (C3), followed by the proteolytic activation of C5. Fragments of these two proteins, including C3a, C3b, iC3b, C3d, C5a and C5b, then proceed to initiate the many processes associated with complement-mediated pro-inflammatory and immunomodulatory effects, including the formation of the membrane attack complex (MAC) (C5b-9) (16). In this process, C3b can be inactivated by complement factor I (CFI) into iC3b (inactive C3b), which is further degraded by a second cleavage into C3dg and then C3d (17) by complement receptor 1 type (CR1/CD35) which acts as a cofactor for CFI (18), and proteases, respectively. C3d binds to complement receptor 2 (CR2/CD21) and augments humoral responses (19). C3b can also bind to CFB and properdin, leading to cleavage by Factor D (FD) and initiation of the amplification loop of complement (20). Complement factor H (CFH) is a major soluble inhibitor of the AP and AP convertase that can also bind tissue sites and exert local control (21), and CFHR4 in contrast promotes the activation of the complement by interacting with C3b and acting as a CFH antagonist. C4, a component of the CP or LP, is cleaved into C4b and C4a by the proteases C1s or MASP-2 (22). C4b in a similar fashion as C3b is proteolytically inactivated by CFI into iC4b and C4d and C4c (23). C4d is deposited on the surface of injured cells and is accepted as a marker of CP/LP activation in renal and cardiac allografts (24, 25), but its role in the synovium after knee injuries is not known. Studies have shown that activated complement components play a central role in joint inflammation in rheumatic diseases (26–29).

Several studies have suggested a key role for the CS in patients with chronic OA and experimental models of this condition (30, 31). In one study of patients with longstanding chronic OA, high levels of complement proteins and activation products were found in the synovial fluid and synovial membrane (31). In this study, using a destabilization of the medial meniscus (DMM) mouse model of OA, markedly lower inflammatory biomarker levels and joint damage were found in mice lacking complement component C5, as compared to wild type (WT) controls (31). In another cartilage blunt trauma model in rabbits, there was deposition of the membrane attack complex (MAC, a.k.a. C5b-9) on the chondrocyte surface (32). CR2-fH, a fusion protein that inhibits activation of C3 and C5 (31, 33), was found in the DMM mouse model to attenuate the development of OA in WT mice (31). Mice deficient in C6, an integral component of the MAC, were also protected against the development of OA and synovitis in the DMM model (31).

In humans, the CS has been shown to be activated in chronic OA and after knee injury (34). Furthermore, after knee injury, this activation is associated with inflammation in synovial fluid as well as with the presence of osteochondral fractures. Interestingly C4d was increased in the SF even several years after knee injury (34). These studies demonstrated an important potential role of complement activation and suggested the use of complement-based therapeutic strategies in OA and PTOA. However, a key gap in our understanding of this process is whether complement is activated in the synovium tissue at the time of ACL injury and MT, and how that relates to infiltration of innate immune cells in the early period. The current study is intended to address these questions by analyzing DSST at the time of ACL reconstruction and meniscectomy. Of particular relevance and significance to these studies is that there are an increasing number of complement inhibitors being developed for the treatment of human diseases which could be applied to prevention of PTOA (35).

The main goal of this study was to examine and compare synovium and ask whether there are specific pathogenic complement proteins and other innate immune cells or immune factors which can contribute to the development of OA after ACL injury and MT. Therefore, we evaluated the synovium for complement proteins and infiltrating immune cells after ACL injury and MT but before ACL reconstructive surgery or meniscectomy, respectively. We compared the presence of complement components and immune cells in these two local but different injuries in the knee joint and asked how two different injuries can lead to one disease i.e., PTOA by using DSST from OA patients. Our hypothesis is that injury specific complement proteins, complement pathways and innate immune cells are elevated or dysregulated in the synovium after ACL injury and MT, which leads to damage to the synovium in a manner that could promote the eventual development of PTOA.

## 2 Material and methods

### 2.1 Histopathology of DSST from ACL and MT injuries

DSST was obtained during ACL reconstruction and medial meniscectomy or medial meniscus repair in ACL or MT patients, respectively, through a minimally invasive knee arthroscopic procedure under local anesthesia by an orthopedic surgeon according to an Institutional Review Board approved protocol. Approximately 6-8 DSST fragments were sorted from a pool of other human tissue such as adipose tissue related to ACL reconstruction (n = 16) or MT meniscectomy (n = 16), or OA before total knee replacement surgery (n = 3) and the size of each synovial tissue was from 0.5mm to 3mm. In the current study, DSST were included from subjects with ACL reconstruction and with both medial and lateral meniscus repair (n = 4) while two DSST samples were from subjects with ACL reconstruction and with lateral meniscus repair only (n = 2). All these DSST samples were then fixed in 10% neutral buffered formalin (NBF) followed by 2x washings at 24 h with 70% Ethanol. The formalin fixed DSST samples were processed for paraffin embedding, followed by sectioning for histopathology and MIHC to assess the presence of various complement proteins and immune cells according to the protocol previously described (36). All DSST samples from ACL and MT were stained using Hematoxylin and Eosin (H & E) and Vimentin followed by examination by a pathologist for quality control purposes. For H & E histopathological analysis, additional DSST samples (n = 4) from MT were included (total n =20), and these samples were not included for complement and immune cells MIHC studies. The following criteria was used to examine vascularity, number of adipocytes, and fibroblast-like synoviocytes: none = 0 blood vessels, 0 adipocytes, and 0 fibroblast-like synoviocytes; low = 1-133 blood vessels, 1-30 adipocytes, and 1-20 fibroblast-like synoviocytes; medium = 134-267 blood vessels, 31-60 adipocytes, and 21-40 fibroblast-like synoviocytes; high = 268-400 blood vessels, 61-90 adipocytes, and 41-60 fibroblast-like synoviocytes. To assess the thickness of the synovial membrane the following criteria was used: none = 0 no synovial membrane present; low = 1-2 layers of synovial membrane cells, medium = 3-4 layers of synovial membrane cells, and high = 5-6 layers of synovial membrane cells. In parallel, from commercially obtained control synovial tissue (n = 4) that was formalin fixed and paraffin-embedded, 2-4 sections on each slide were processed from cadaveric donors knee joints (ST-1420 (Caucasian, male, 32); ST-1443 (Caucasian, male, age 49); ST-1429 (Caucasian, female, 44); ST-1358 (Caucasian, male, 53) with no history of opportunistic infections, rheumatoid arthritis (RA) and OA (Articular Engineering, IL). Serology tests from these control donors were negative for HIV-1, Hepatitis C antibody, and Hepatitis B antigen. For comparison of complement proteins and immune cells, some of the DSST samples (n = 3) were also obtained as a surgical discard from OA patients (ages 43, 60, 55) after total knee replacement surgery.

### 2.2 Multiplexed immunohistochemistry of DSST for complement proteins

DSST from ACL (n = 16) and MT (n =16) patients obtained during reconstructive surgery or before meniscectomy, respectively, were used to simultaneously detect specific complement proteins as complement panel 1, according to our published method (36). Briefly, complement panel 1 included MIHC for detecting C3c, CFH, CFB, CFHR4, FCN3, MBL2 and C5b-9 (aka MAC) in the synovium (36). For current studies, we also standardized another complement panel 2 using DSST from ACL (n = 9) and MT (n = 12), which included MIHC for detecting C3d, C4d, C5/C5b, C3AR1, C5AR1, CD35 (aka CR1), CD21 (aka CR2), CD55 (aka DAF, decay-accelerating factor), and CFP. For standardization of complement panel 2, we used the following antibodies as well as the specific antigen retrieval (AR) procedures for MIHC. For C3d, the antigen retrieval used was 10mM Sodium Citrate pH 6.0 for 10 min at 110°C using Ventana Stainer. The dilution of the primary C3d antibody (LSBio) used was 1:100 and incubated for 32 min at 37°C. Detection of C3d was done using Ventana I-VIEW DAB, secondary replaced with Rabbit ImmPress polymer (Vector Labs), enzyme replaced with Rabbit ImmPress polymer at 50% strength (Vector Labs). For C4d, Borg high 9.5 pH solution (Biocare Medical) was used for 10 min at 110°C in the NxGen Decloaker (pressure cooker) (Biocare Medical). The dilution of primary C4d antibody (American Research product) used was 1:10000 and incubated for 32 min at 37°C in the benchmark XT IHC Stainer. Detection of C4d was done with the UltraView DAB kit from Ventana/Roche. For C5/C5b, Borg solution pH 9.5 (Biocare Medical) was used for 10 min at 110°C followed by cooling for 10 min at room temperature. The dilution of primary C5/C5b antibody used was 1:200 and incubated for 32 m in at 37°C on a benchmark XT IHC stainer. Detection of C5/C5b was done with Ventana UltraView DAB universal polymer detection from Ventana/Roche, using the Ventana Benchmark XT. For C3aR1, 10 mM Sodium Citrate solution pH 6.0 for 10 min at 110°C. The dilution of the primary C3aR1 antibody (LsBio) used was 1:100 and incubated for 32 min at 37°C. Detection of C3aR1 was done using Rabbit modified I-View. For C5aR1, similarly 10 mM Sodium Citrate solution pH 6.0 for 10 min at 110°C, antigen retrieval procedure, was used as mentioned above. The dilution of the primary C5aR1 antibody (Abcam) (rabbit polyclonal) used was 1:200 and incubated 32 min. at 37°C. The detection of C5aR1 was done for 10 min at 37°C using Rabbit modified I-View. For CD35, antigen retrieval was done using 10 mM Sodium Citrate pH 6.0 with incubation for 10 min at 110°C. The dilution of primary CD35 antibody (aka CR1) (Abcam) (rabbit polyclonal) used was 1:200 incubated for 32 min at 37°C. The detection of CD35 was done using Rabbit modified I-View. For CD21, 10 mM Sodium Citrate pH 6.0 with incubation for 10 minutes at 110°C. The dilution of the primary CD21 (aka CR2) antibody (Abcam) used was 1:200 and incubated for 32 min at 37°C. The detection of CD21 was done using Rabbit modified I-View. For CD55, 10 mM Sodium Citrate pH 6.0 with incubation for 10 min at 110°C then cooled for 10 min at room temperature. The dilution of the primary CD55 (aka DAF) antibody (Abcam) used was 1:200 and incubated for 16 min at 37°C. The detection of CD55 was done using the UltraView Universal detection kit. For CFP, the antigen retrieval procedure used was BORG high pH 9.5 10 min at 110°C in the pressure cooker. The dilution of the primary anti-Properdin/CFP rabbit anti-human polyclonal antibody (aa35-84) (LS Biosciences, Inc.) was 1:200 and incubated for 32 min at 37°C. The detection of CFP (Properdin) was done using the Ventana OptiView DAB kit. The counterstain was done with Harris Hematoxylin with 2 min incubation. Various human tissue samples (surgical discard) such as OA synovium, normal kidney, transplant rejected kidney, lungs, liver, and spleen were used as negative and positive controls to examine the specificity of the complement antibodies for optimization. All complement related MIHC staining was performed using the Akoya Biosciences detection kit and multispectral imaging using the Vectra Polaris systems. MIHC images from DSST were scanned to obtain the percentage of cells expressing complement proteins as described below.

### 2.3 Quantitative measurements of complement proteins from ACL and MT DSST

After scanning and imaging of MIHC images from ACL and MT synovium, all data were exported from the image analysis software (InForm) according to our published studies (36). The frequency of positive cells for each complement protein from each synovial section was calculated by dividing the number of cells with positive staining by the total number of cells in each region. The average frequency was then calculated across all the regions imaged on each slide. The software identifies each individual cell using 4′,6-diamidino-2-phenylindole (DAPI) staining nuclei. Parameters were set to define the nuclear size, intensity, and shape. A cytoplasmic area was drawn around each nucleus. In the scoring function, a threshold is set for each marker depending on the range of signals seen across the images. The percentage of cells that are above the threshold are considered positive and quantified. In MIHC scanning analysis, an individual cell can be positive for more than one category of marker. For example, there may be some overlap between two markers that one would not expect, caused potentially by two cells being on top of one another or co-expressing two markers. A false color-coding scheme was used to identify each complement protein, receptor and immune cell.

### 2.4 Multiplexed immunohistochemistry of DSST from ACL and MT for immune and inflammatory cells

A panel of antibodies and antigen retrieval procedures (36) were used to detect the presence of immune and inflammatory cells in the DSST from ACL injury (n = 10), MT (n = 16) and control (n = 4) injuries using MIHC followed by quantitative imaging analysis, as described before (36). Due to the availability of limited DSST from OA patients no MIHC analysis was done for various immune cells. When we included and reanalyzed DSST data from six additional subjects with both ACL reconstructive surgeries as well as MT- related meniscectomy along with nine ACL reconstructive surgeries subjects alone, as mentioned in Methods, we found there were still significant differences related to all parameters compared with DSST from MT related meniscectomy alone again confirming that ACL injury affected the synovium more severely than the MT (data not shown).

### 2.5 Statistical analyses

The differences between the percentage of complement protein positive cells from the ACL and MT synovium using MIHC quantitative imaging data were compared by using both student t-test and Mann and Whitney test a.k.a. Wilcoxson-Mann-Whitney test. All p-values for significance were confirmed by using Mann and Whitney test, a nonparametric test. All data were plotted using GraphPad Prism 5. Data were expressed as Mean ± SEM with p-values < 0.05 were considered significant.

## 3 Results

### 3.1 Characteristic of ACL and MT patients

**Figure 1:**
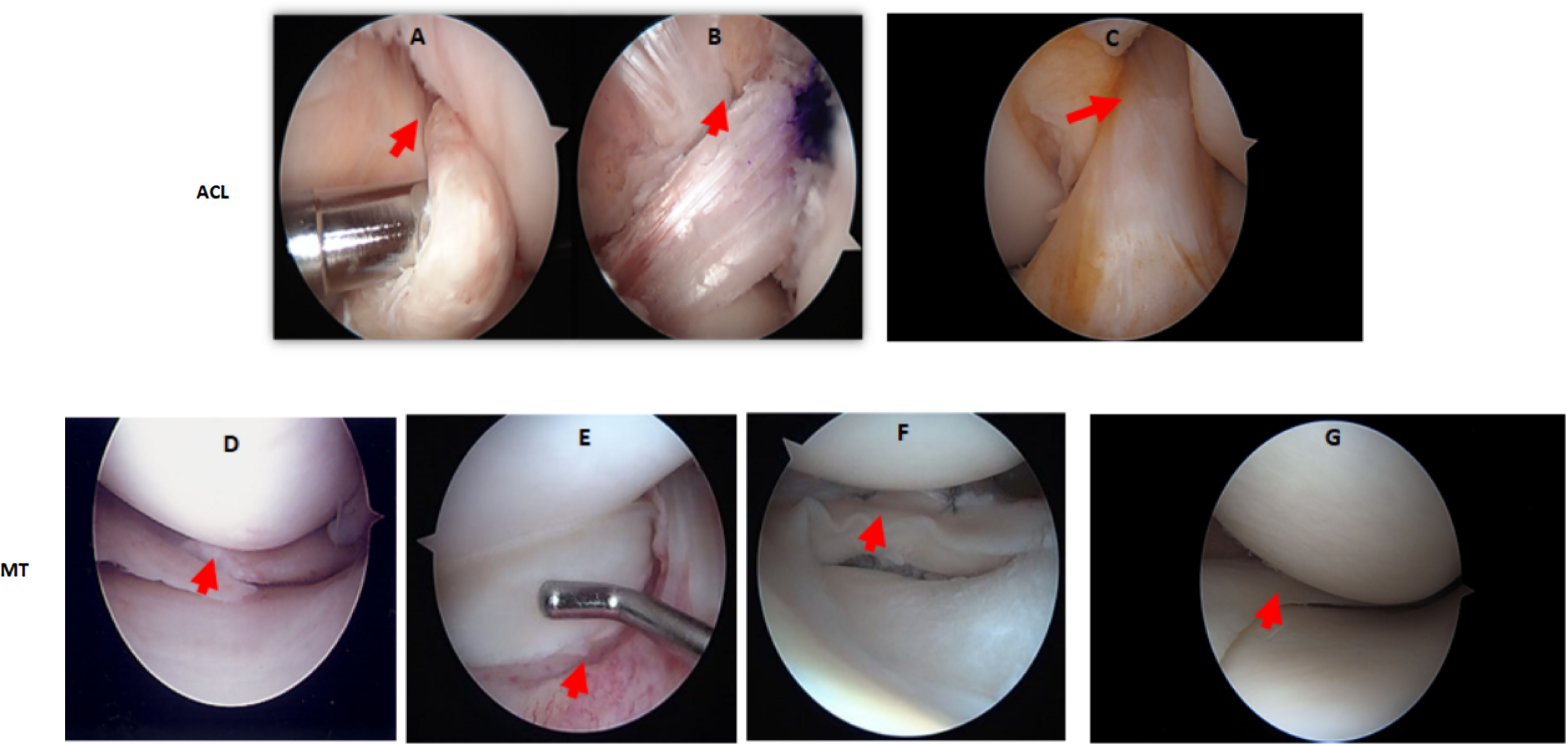
Arthroscopic images of the knee joint ACL injury before and after reconstructive surgery, or of the MT before and after meniscectomy. **(A)** ACL injury before reconstructive surgery. **(B)** ACL after reconstructive surgery. **(C)** Normal ACL with no injury and no reconstructive surgery. **(D)** Medial meniscus radial tear before meniscectomy **(E)** Lateral meniscus bucket handle tear before meniscectomy. **(F)** Lateral meniscus bucket handle tear after meniscectomy. **(G)** Normal medial meniscus with no injury and no meniscectomy. Knee arthroscopy was done by an Orthopedic surgeon and images were taken before and after surgery. In arthroscopic images of ACL injury and post reconstructive surgery, and of MMT/LMT and post meniscectomy, relevant features are indicated by a red arrow.

Demographic characteristics of patients with ACL and MT are summarized in **Table 1**. Additional characteristics of each patient including sex, age, body mass index (BMI), race, ethnicity, specific diagnosis, surgical procedure, and the limb from which tissue was obtained are shown in **Tables 2**, **3**. Although there were no major differences in the age and BMI, many BMI were in the overweight and obesity ranges (**Tables 2**, **3**). There were more ACL and MT DSST samples from females than males, as all MIHC studies were carried out blindly, with the recruitment population restricted by the availability of DSST. Representative clinical images of the ACL before (**Figure 1A**) and after reconstruction (**Figure 1B**), normal ACL (**Figure 1C**) and various types of MT before meniscectomy (**Figure 1D**), after meniscectomy (**Figures 1E, F**) and normal medial meniscus with no tear and no meniscectomy (**Figure 1G**) are shown.

**Table 1:** Characteristics of ACL and MT Synovial Tissue Patients.

DSST	ACL	MT
N	16	16
Age: mean ± SD	36.1 ± 10.8	50.2 ± 10.2
Gender: % female	62.5	81.3
Race: % Caucasian	81.3	87.5
Ethnicity: % non-Hispanic	93.8	93.8
BMI: mean ± SD	25.7 ± 4.2	32.4 ± 20.0
Orientation: % right	75	62.5

DSST, Discarded surgical synovial tissue; ACL, Anterior cruciate ligament; MT, Meniscus Tear; BMI, Body mass index.

**Table 2:** Characteristics of ACL^1^ Synvoial Tissue Patients.

DSST						Joint	Primary	Secondary	Procedure 1	Procedure 2
N	Sex	Age	BMI^2^	Race	Ethnicity	Side	DX	DX		
1.0012-2	F	29	29.67	Black	Non-Hispanic	Right	ACL		Arthroscopy	ACL reconstruction
2.0014-2	M	44	25.88	Caucasian	Non-Hispanic	Right	ACL	Medial and lateral meniscus tear	Arthroscopy	ACL reconstruction and both medial and lateral meniscus sx
3.0020-2	F	39	22.32	Caucasian	Non-Hispanic	Right	ACL		Arthroscopy	
4.0023-2	F	41	22.23	Caucasian	Non-Hispanic	Right	ACL		Arthroscopy	ACL reconstruction
5.0032-2	F	27	32.42	Caucasian	Non-Hispanic	Left	ACL		Arthroscopy	ACL reconstruction
6.0039-2	F	36	21.47	Caucasian	Non-Hispanic	Left	ACL	Lateral meniscus tear	Arthroscopy	ACL reconstruction and lateral meniscus sx
7.0044-2	M	28	20.9	Caucasian	Non-Hispanic	Right	ACL		Arthroscopy	ACL reconstruction
8.0045-2	M	33	23.5	Caucasian	Non-Hispanic	Right	ACL		Arthroscopy	ACL reconstruction
9.0054-2	F	40	28.67	Caucasian	Non-Hispanic	Left	ACL		Arthroscopy	ACL reconstruction
10.0055-2	F	12	20.74	Caucasian	Non-Hispanic	Right	ACL		Arthroscopy	ACL reconstruction
11.0080-2	M	26	28.8	Asian	Non-Hispanic	Right	ACL		Arthroscopy	ACL reconstruction
12.0086-2	F	42	23.42	Caucasian	Non-Hispanic	Right	ACL	Medial and lateral meniscus tear	Arthroscopy	ACL reconstruction and both medial and lateral meniscus sx
13.0119-2	F	51	24.34	Caucasian	Non-Hispanic	Right	ACL	Medial and lateral meniscus tear	Arthroscopy	ACL reconstruction and lateral meniscus sx
14.0120-2	M	47	34.74	Caucasian	Unknown	Right	ACL	Medial and lateral meniscus tear	Arthroscopy	ACL reconstruction and both medial and lateral meniscus sx
15.0124-2	M	29	25.62	Caucasian	Non-Hispanic	Left	ACL	Lateral meniscus tear	Arthroscopy	ACL reconstruction and lateral meniscus sx
16. 0126-2	F	54	26.23	Black	Non-Hispanic	Right	ACL		Arthroscopy	ACL reconstruction

^1^Anterior cruciate ligament.

^2^ Body mass index.

DSST, discarded surgical synovial tissue; Sx, surgery.

**Table 3:** Characteristics of MT^1^ Synovial Tissue Patients.

DSST						Joint	Primary DX	Secondary DX	Procedure 1	Procedure 2
N	Sex	Age	BMI^2^	Race	Ethnicity	Side				
**1.0019-2**	M	57	23.99	Caucasian	Non-Hispanic	Right	MMT	Synovitis	Arthroscopy	Debridement
**2.0038-2**	F	36	19.37	Caucasian	Non-Hispanic	Right	MMT		Arthroscopy	Meniscectomy
**3.0042-2**	F	59	25	Caucasian	Non-Hispanic	Right	MMT		Arthroscopy	Medial Meniscectomy
**4.0048-2**	F	54	33.28	Caucasian	Non-Hispanic	Right	MMT	Medial Meniscus tear	Arthroscopy	Synovectomy
**5.0058-2**	F	52	24.96	Caucasian	Non-Hispanic	Right	MMT		Arthroscopy	Lateral Meniscectomy
**6.0066-2**	M	62	25.1	Caucasian	Non-Hispanic	Left	MMT		Arthroscopy	Medial Meniscectomy
**7.0089-2**	F	45	104.26	Caucasian	Non-Hispanic	Left	MMT		Arthroscopy	Debridement
**8.0100-2**	F	45	23.76	Caucasian	Non-Hispanic	Left	MMT		Arthroscopy	Meniscectomy
**9.0103-2**	F	50	23.03	Caucasian	Non-Hispanic	Right	MMT		Arthroscopy	Debridement
**10.0106-2**	F	64	33.68	Caucasian	Non-Hispanic	Right	MMT		Medial Meniscectomy	Lateral Meniscectomy
**11.0116-2**	F	61	25	Caucasian	Non-Hispanic	Right	MMT		Arthroscopy	Partial Lateral Meniscectomy
**12.0127-2**	F	49	33.75	Multiracial	Non-Hispanic	Right	MMT	Synovitis	Arthroscopy	Partial Medial Meniscectomy
**13.0130-2**	F	46	40.66	Caucasian	Non-Hispanic	Left	MMT		Arthroscopy	Partial Medial Meniscectomy
**14.0133-2**	M	57	29.99	Caucasian	Non-Hispanic	Left	MMT	Synovitis	Arthroscopy	Partial Medial Meniscectomy
**15. 0135-2**	F	38	22.1	Caucasian	Non-Hispanic	Left	MMT	Lateral Meniscus Tear	Arthroscopy	Partial Medial Meniscectomy
**16. 0136-2**	F	28	30.12	Other	Hispanic	Right	MMT		Arthroscopy	Lateral Meniscectomy

^1^ Meniscus tear.

^2^ Body mass index.

DSST, discarded surgical synovial tissue.

### 3.2 Histopathology of DSST from ACL and MT

**Figure 2:**
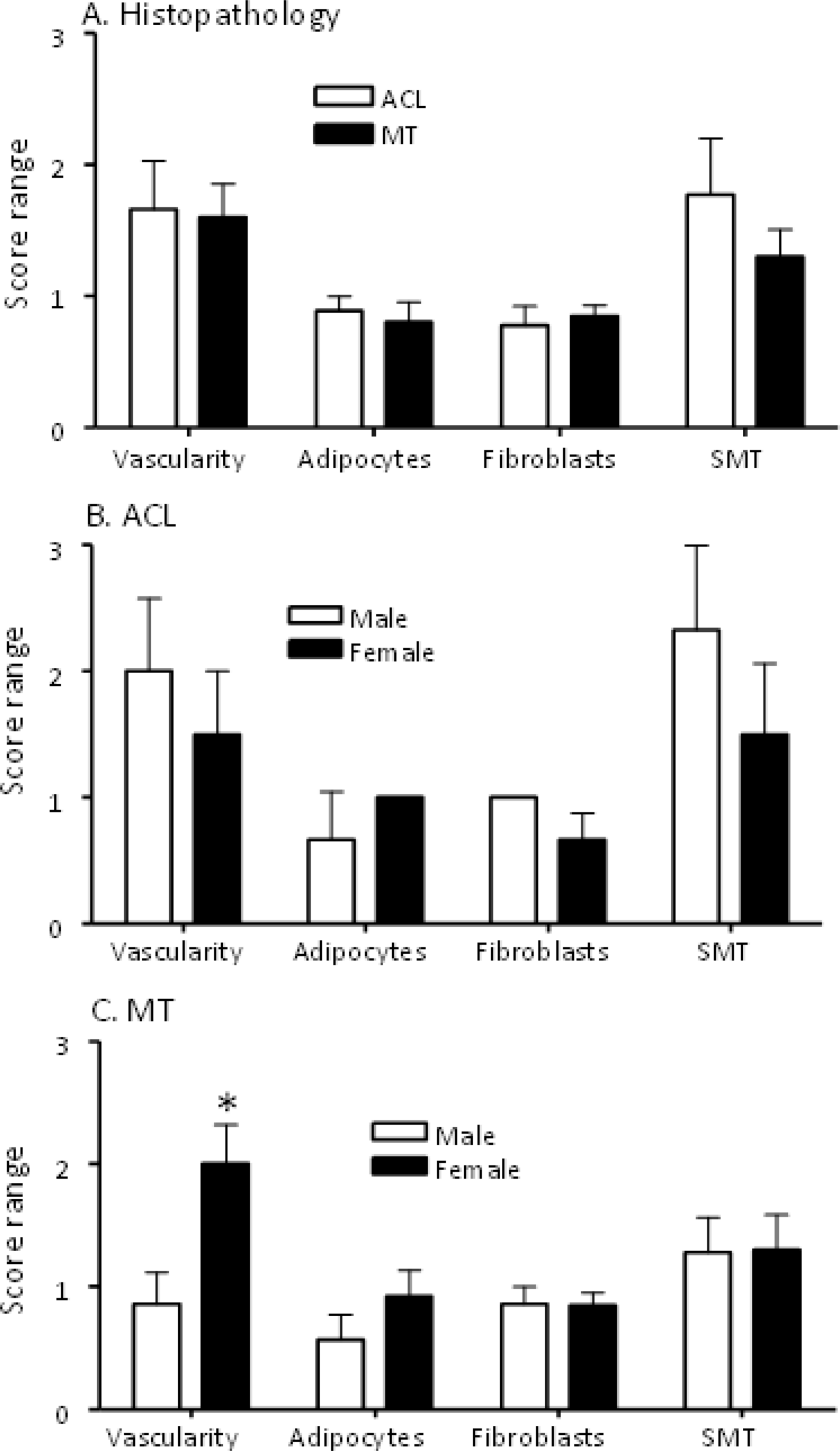
Comparing histopathological characteristics of DSST from ACL injury and MT patients. All DSST were staining with Hematoxylin and Eosin (H & E) followed by counting in each section of blood vessels, adipocytes, fibroblast-like synoviocytes and the numbers of cell layers present in the synovium membrane. **(A)** Synovium from ACL and MT patients showing no differences in the presence of vascularity, adipocytes, fibrosis and synovial membrane thickness. **(B)** Synovium from male and female ACL patients showing no differences in vascularity, adipocytes, fibrosis and synovial membrane thickness. **(C)** Synovium from female MT patients compared with male showing increase in vascularity with no differences in adipocytes, fibrosis and synovial membrane thickness. SMT = synovial membrane thickness. ACL DSST n = 9. MT DSST n = 20. Male ACL DSST n = 3 and female ACL DSST n = 6. Male MT DSST n = 7 and female MT DSST n = 13. All DSST were included in the final analysis for reproducibility and variability. Data are shown as mean ± SEM. *p < 0.05 considered significant.

To examine the quality of DSST obtained, H & E staining was performed on each synovial fragment from all ACL and MT patients included in this study (**Tables 2**, **3**). For this analysis, DSST from ACL and MT were blindly scored using a scale from 0-3 for vascularity, density of adipocytes, fibrosis, and synovial membrane thickness (SMT) (**Figure 2**), as described in the Methods. A subset of the ACL and MT DSST samples were also stained for Vimentin as an internal quality control stain; it was uniformly present confirming that the architecture and integrity of the tissue was intact (data not shown).

**Figure 3:**
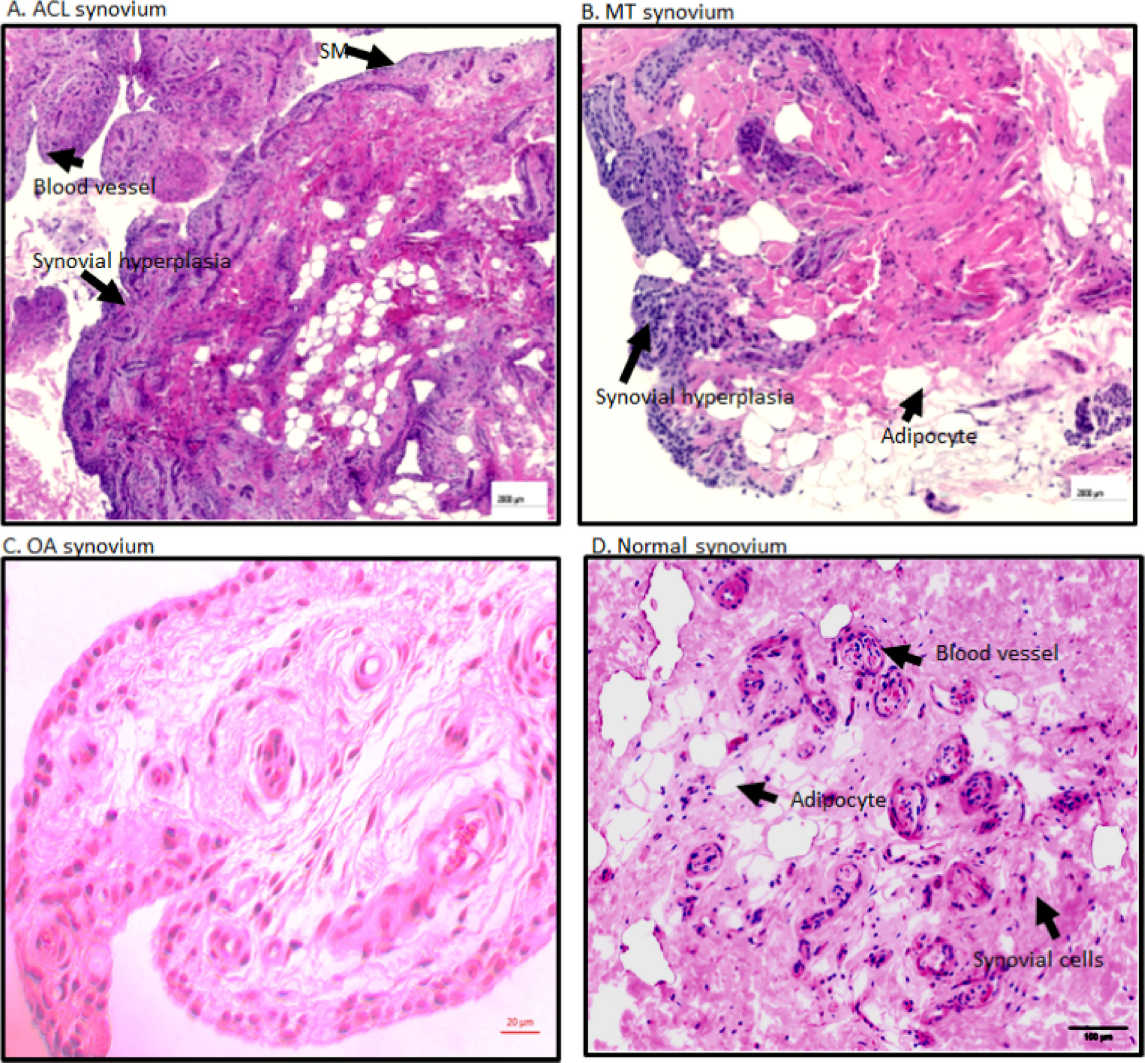
Representative H & E histopathology images of the DSST after ACL injury and MT. **(A)** DSST after ACL injury. **(B)** DSST after MT. **(C)** OA DSST before knee replacement surgery. **(D)** Normal DSST with no RA and no OA. Blood vessels, adipocytes, and synovial membrane have been marked with a black arrow. These ACL and MT representative images have been selected from ACL DSST n = 9. MT DSST n = 20. OA DSST n = 3. Normal DSST n = 4. For quality control two sections from each DSST were analyzed using H & E staining. Scale = 2000µm except for **(C)** scale = 20um.

Overall, there were no significant differences between ACL and MT synovial tissue regarding vascularity, number of adipocytes, fibrosis and SMT (**Figures 2A, B**). However, further analysis of MT synovial tissue showed that there was significantly (p < 0.028) more vascularity in females than males (**Figure 2C**), suggesting that sex-specific differences exist in the synovial tissue after MT injury. Each DSST from ACL, MT, OA and normal control were examined histologically (**Figure 3**). Histologically control DSST obtained appeared normal although from cadaver donors (**Figure 3D**) therefore an appropriate normal synovium. Representative H & E images of the ACL, MT, OA and normal DSST used for assessing the above parameters are shown (**Figures 3A–D**).

### 3.3 Complement injury-related markers in the DSST from ACL and MT

**Figure 4:**
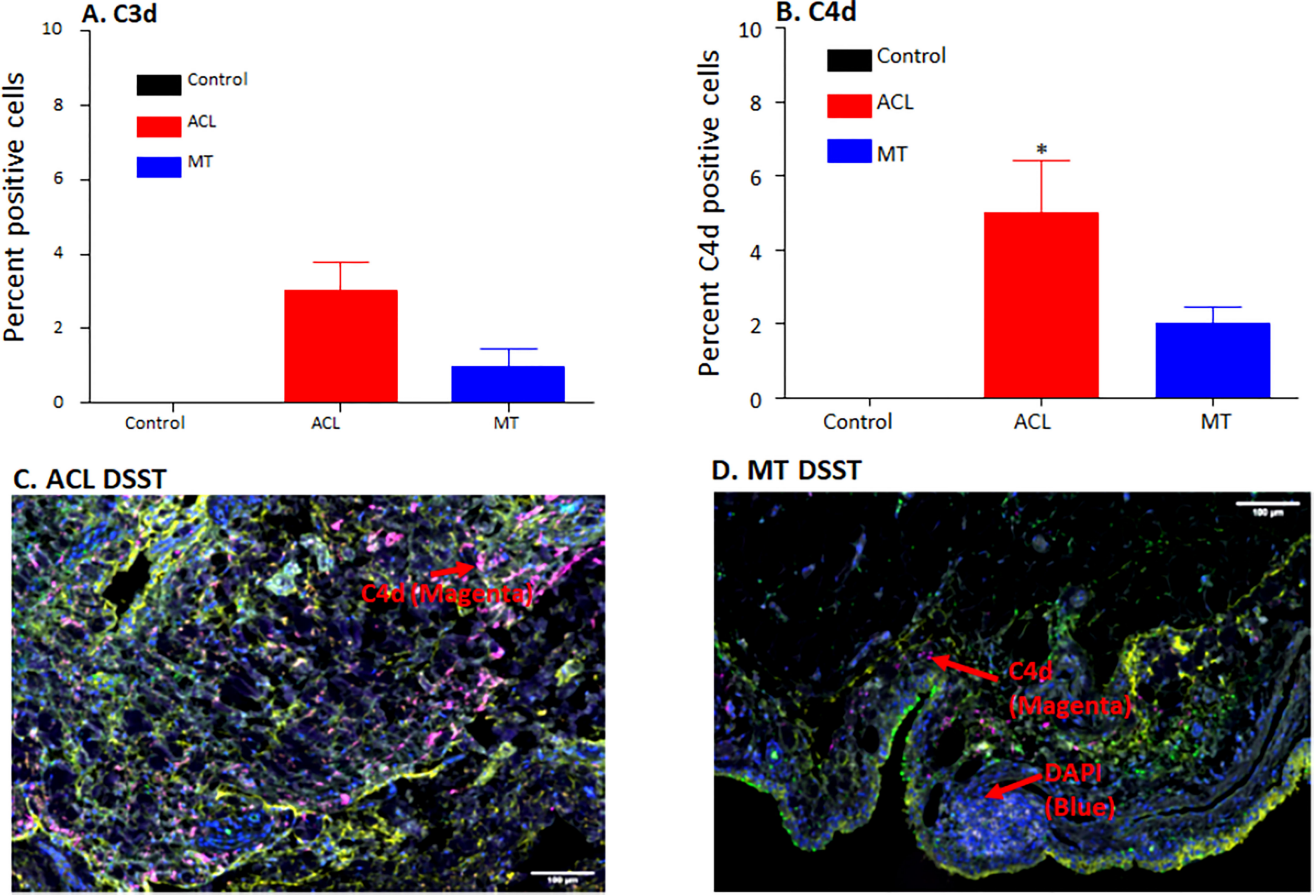
Comparing using MIHC percentage of synovial cells expressing C3d and C4d and representative composite images of MIHC comparing C4d in the DSST obtained from ACL injury and MT at the time of reconstructive surgery and meniscectomy, respectively. **(A)** C3d positive cells in control (black color), ACL (red color) and MT (blue color) synovium. **(B)** C4d positive cells in control (black color), ACL (red color) and MT (blue color) synovium. Control synovial tissue were with no RA and no OA, n = 3. ACL n = 9. MT n = 12. Two sections from each biopsy were analyzed. Data are shown as Mean ± SEM. *p < 0.05 considered significant. **(C)** A composite image from ACL synovium showing the presence of more C4d (magenta color). **(D)** A composite image from MT synovium showing the presence of less C4d (magenta color). Each complement protein has been shown using false color coding, including Magenta = C4d, Orange = C3d, Yellow = DAF and DAPI = blue. A red arrow shows the presence of a complement positive cell. A minimum of three composite images were generated using a single DSST with 2 sections from each ACL (n = 9) and MT (n = 12) patient. Scale bar = 100µm.

DSST from patients with ACL injury and MT were examined for the presence of the C3d and C4d split products that are covalently attached in tissues and derived from C3 and C4, respectively (**Figure 4**). There were no differences in the percentage of synovial cells expressing C3d (p = 0.20) in the DSST from both ACL injury and MT; however, there was a significant (p < 0.009) increase in the percentage of C4d positive cells in the ACL synovium compared with the MT synovium (**Figures 4A, B**). Minimum baseline levels of C3d and C4d were present as expected in the control synovial tissue (**Figures 4A, B**). Due to limited availability of DSST from OA, MIHC for C3d and C4d expressing synovial cells were not performed. A representative composite image of C4d (magenta color) from ACL and MT synovial tissue is shown (**Figures 4C, D**). As C4d is an injury related marker and binds to the site of complement activation, these data suggest that ACL injury affects the synovium through CP/LP activation more than found with MT. Thus, ACL injury might be differentially activating the complement system pathways as compared to MT.

### 3.4 Alternative pathway dysregulation in the synovium after ACL injury

**Figure 5:**
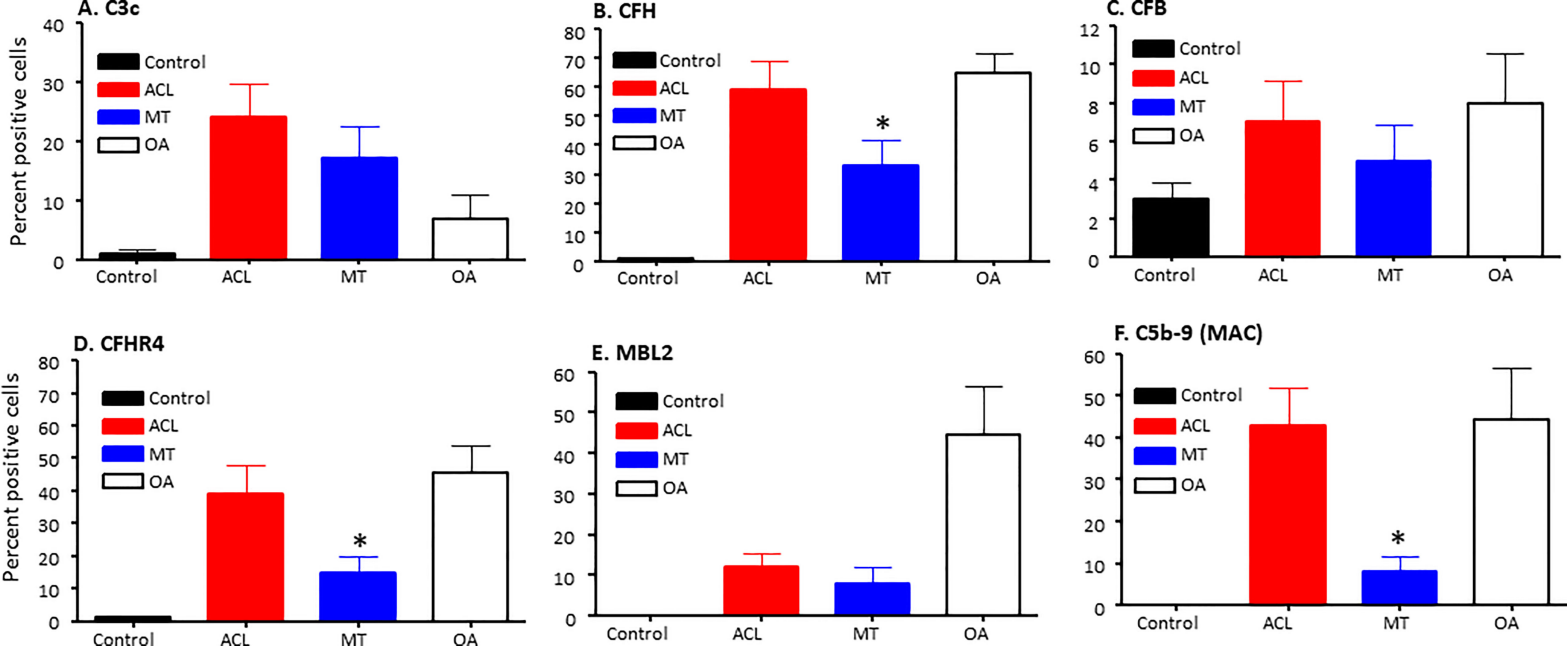
Complement alternative pathway, its components and membrane attack complex in ACL synovium compared with MT synovium obtained at the time of reconstructive surgery or meniscectomy, respectively. Percentage of complement positive synovial cells using MIHC have been shown using imaging analysis. **(A)** C3c **(B)** CFH **(C)** CFB **(D)** CFHR4 **(E)** MBL2 **(F)** C5b -9 (MAC). Control synovial tissue were with no RA, no OA no opportunistic infections (n = 4). ACL n = 10 for C3c, CFH, CFB, CFHR4 and C5b-9. MT n = 16 for C3c, CFH, CFB, CFHR4, MBL and C5b-9. OA n = 3 for C3c, CFH, CFB, CFHR4, MBL and C5b-9. Two sections from each DSST were analyzed. Data are shown as Mean ± SEM. *p < 0.05 considered significant.

**Figure 6:**
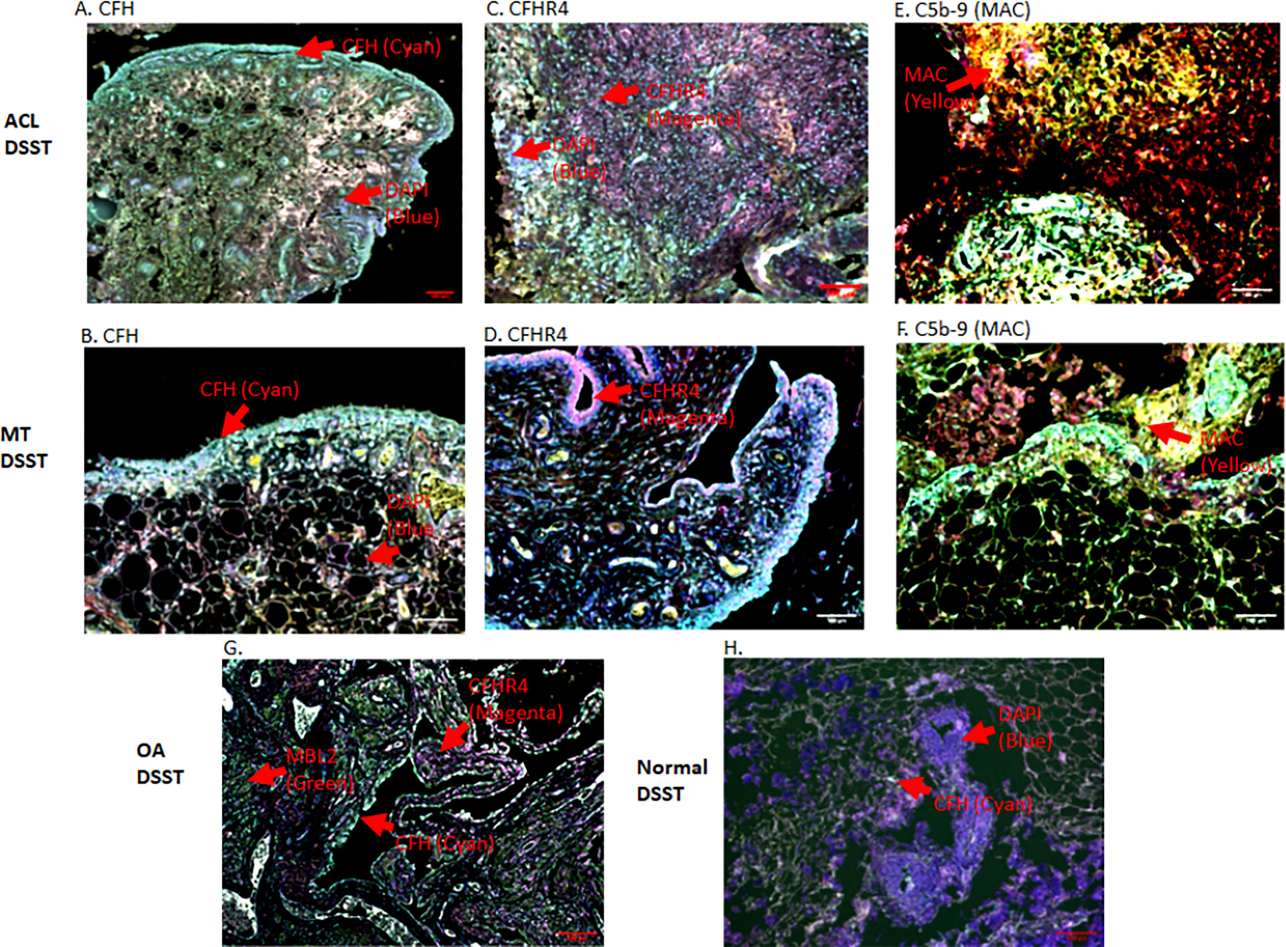
Representative composite images of MIHC showing and comparing the presence of CFH, CFHR4 and C5-9 (MAC) expressing cells in the DSST obtained after ACL and MT injuries but at the time of reconstructive surgery and meniscectomy, respectively. **(A, B)** A significantly high levels of CFH expressing synovial cells in ACL (left first panel) vs. MT (left second panel) synovium. **(C, D)** Higher percentage of CFHR4 expressing synovial cells in ACL (first center panel) vs. MT synovium (second center panel **(E, F)** A significantly high percentage of MAC expressing synovial cells in ACL (first right panel) vs. MT synovium (second center right panel). **(G)** OA DSST (third bottom left panel). **(H)** Normal DSST (third bottom right panel). Each complement protein has been shown using false color coding, including Cyan = CFH, Magenta = CFHR4, MAC = Yellow, MBL2 = Green, CFB = Pink, FCN3 = White, and DAPI = blue. A red arrow shows the presence of a complement positive cell. A minimum of three composite images were generated using a single DSST with 2 sections from each ACL (n = 10) and MT (n = 16) patient. Scale bar = 100µm.

To specifically examine the potential role of the AP in DSST from ACL and MT, which as noted above was an important contributor to experimental OA (31), we examined various components and regulatory proteins of the AP and LP including C3c, CFB, CFP, CFH, CFHR4, MBL2 and also the terminal pathway, C5b-9 (MAC) (**Figure 5**). No significant differences were seen in the ACL synovium compared with the MT synovium for the presence of cells staining positively for C3c, CFB (**Figures 5A, C**) and CFP (data not shown). Interestingly, CFH and CFHR4 proteins were present at significantly (p < 0.045 and p < 0.015, respectively) higher levels in the ACL synovium as compared to MT synovium (**Figures 5B, D**). These data suggest that there is more dysregulation of the AP after ACL injury than the MT, since CFH and CFHR4 counterbalance each other to control the level of AP activation. A minimum baseline of C3c, CFH, CFB, CFHR4, MBL2 and C5b-9 (MAC) were present in the control synovium (**Figures 5A–F**) and CFP (data not shown). Interestingly a significant (p < 0.0007) higher levels of MBL2 expressing cells were found in OA DSST (**Figure 5E**). The higher expression of MBL2 in OA compared with ACL and MT DSST indicate that specific LP components might be playing a role in the late stage of the disease. Representative images of the CFH (cyan color) and CFHR4 (magenta color) proteins on the surface of ACL and MT synovium are shown (**Figures 6A–D**). These data suggest increased levels of activation of the AP are also consistent with the significantly (p < 0.035) higher percentage of C5b-9 (MAC) positive cells in the ACL DSST compared with the MT DSST (**Figures 6E, F**). In parallel and consistently, a significantly higher expression of C5/C5b was found in ACL derived synovial fragments compared with the MT synovial fragments (data not shown). A representative image of the ACL and MT synovium showing the presence of C5b-9 (MAC) (yellow color) is shown (**Figures 6E, F**). Representative composite images of the OA and normal control DSST have been shown (**Figures 6G, H**).

### 3.5 Comparing the expression of complement receptors in the DSST from ACL and MT

**Figure 7:**
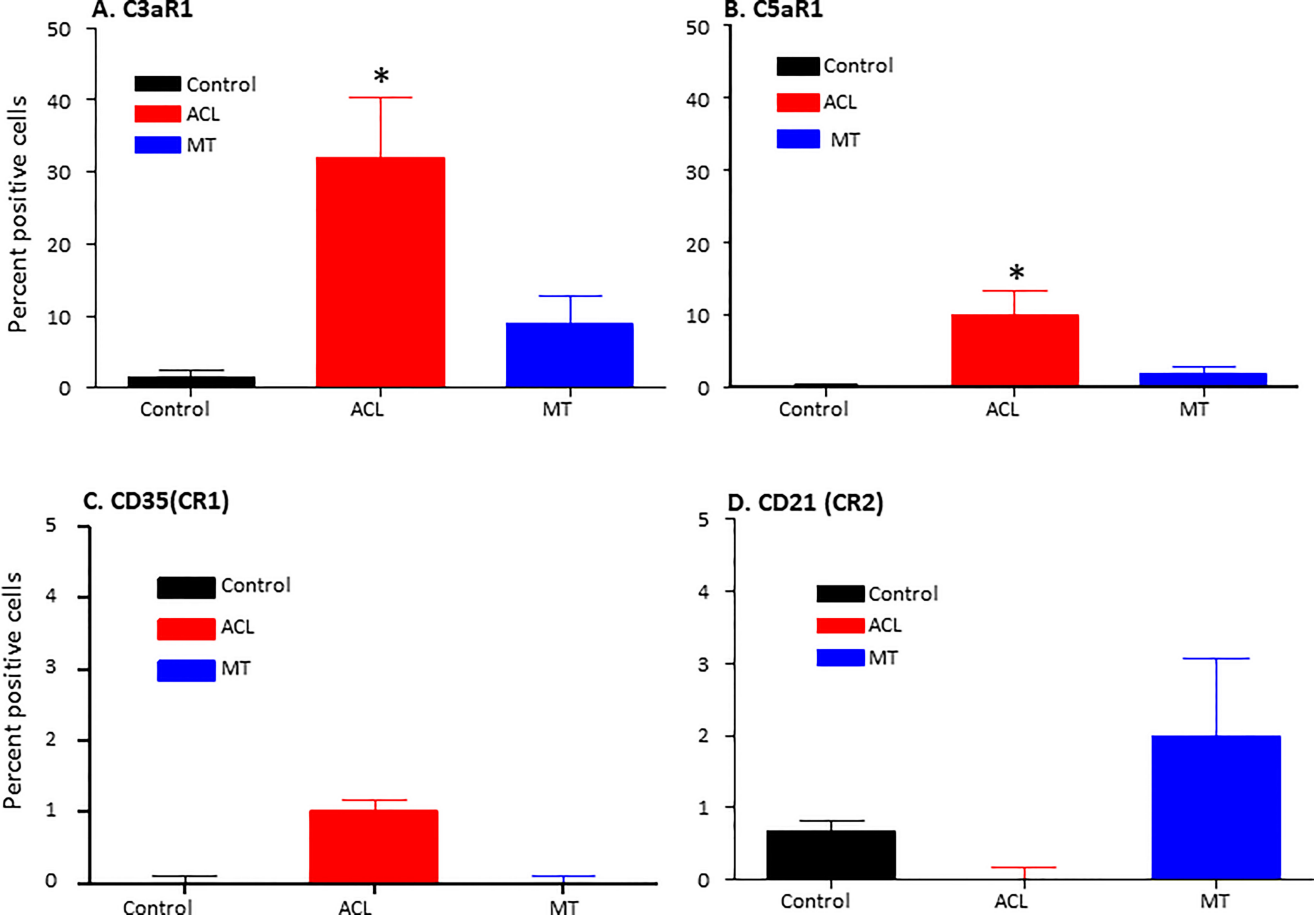
Comparison of complement receptors using MIHC in the DSST after ACL and MMT injuries at the time of reconstructive surgery and meniscectomy respectively. Percentage of positive cells in control (black color), ACL (red color) and MT (blue color) DSST have been shown. **(A)** C3aR1 **(B)** C5aR1 **(C)** CD35 (CR1) **(D)** CD21 (CR2). ACL n = 9 for C3aR1, C5aR1, CR1 and CR2. MT n = 12 for C3aR1, C5aR1, CR1 and CR2. Two sections from each DSST were analyzed. Data are shown as Mean ± SEM. *p < 0.05 considered significant. Normal control n = 4.

**Figure 8:**
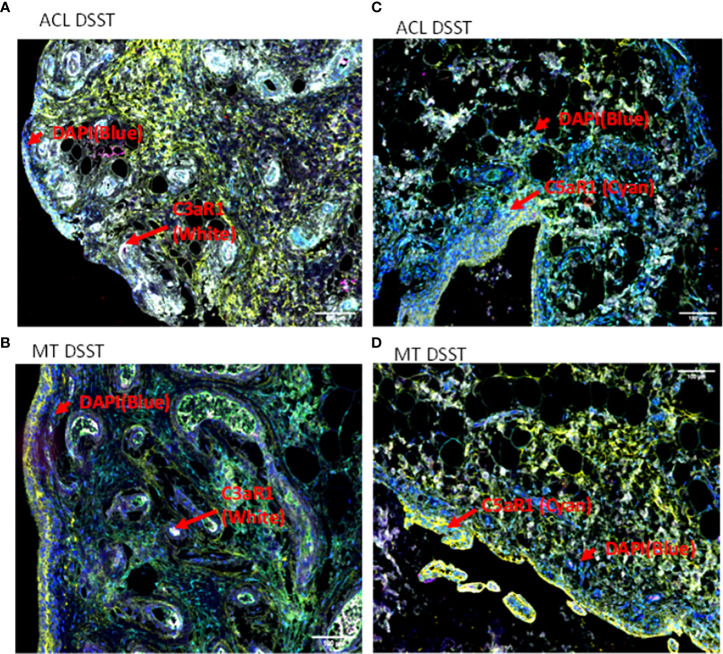
Representative composite images of MIHC comparing the presence of complement receptors in the DSST from ACL and MT obtained at the time of reconstructive surgery and meniscectomy, respectively. **(A, B)** Composite images from ACL and MT DSST respectively showing the presence of C3aR1 (Top and bottom left). **(C, D)** Composite images from ACL and MT DSST, respectively, showing the presence of C5aR1 (Top and bottom right). Each complement receptor or protein has been shown using false color coding, including specifically expressed at a higher levels surrounding blood vessels White = C3aR1, Cyan = C5aR1, Yellow = DAF and DAPI = blue. A red arrow shows the presence of a complement positive cell. A minimum of three composite images were generated using a single DSST with 2 sections from each ACL (n = 9) and MT (n = 12) patient. Scale bar = 100µm shown either at the top or at the bottom right-hand side of each composite image.

To examine potential mechanisms by which the complement system could play a causal role in the development of synovial inflammation, we also examined in the DSST from ACL injury and MT for the presence of informative complement receptors (i.e., C3aR1 and C5aR1) to which the activation-generated components of the complement system, designated C3a and C5a, respectively, bind (**Figure 7**). A significantly higher percentage of C5aR1 and C3aR1 (p < 0.013) (p < 0.024) expressing synovial cells were present in the ACL compared with the MT synovium (**Figures 7A, B**). Representative composite images regarding the presence of C3aR1 and C5aR1 receptors in synovium from ACL and MT are shown (**Figures 8A, B**). A high expression of specifically C3aR1 was noticed surrounding blood vessels in the ACL DSST (**Figure 8A**). In contrast, there were no differences in the percentage of synovial cells expressing CR1/CD35 (p = 0.25) and CD21/CR2 (p = 0.33) both in DSST from ACL injury and MT (**Figures 7C, D**). MIHC representative composite images of the synovial tissue from ACL injury (**Figures 8A, C**) and MT (**Figures 8B, D**) are shown for the presence of C3aR1 and C5aR1 receptors.

### 3.6 Comparing immune and infiltrating cells in the DSST from ACL injury and MT

**Figure 9:**
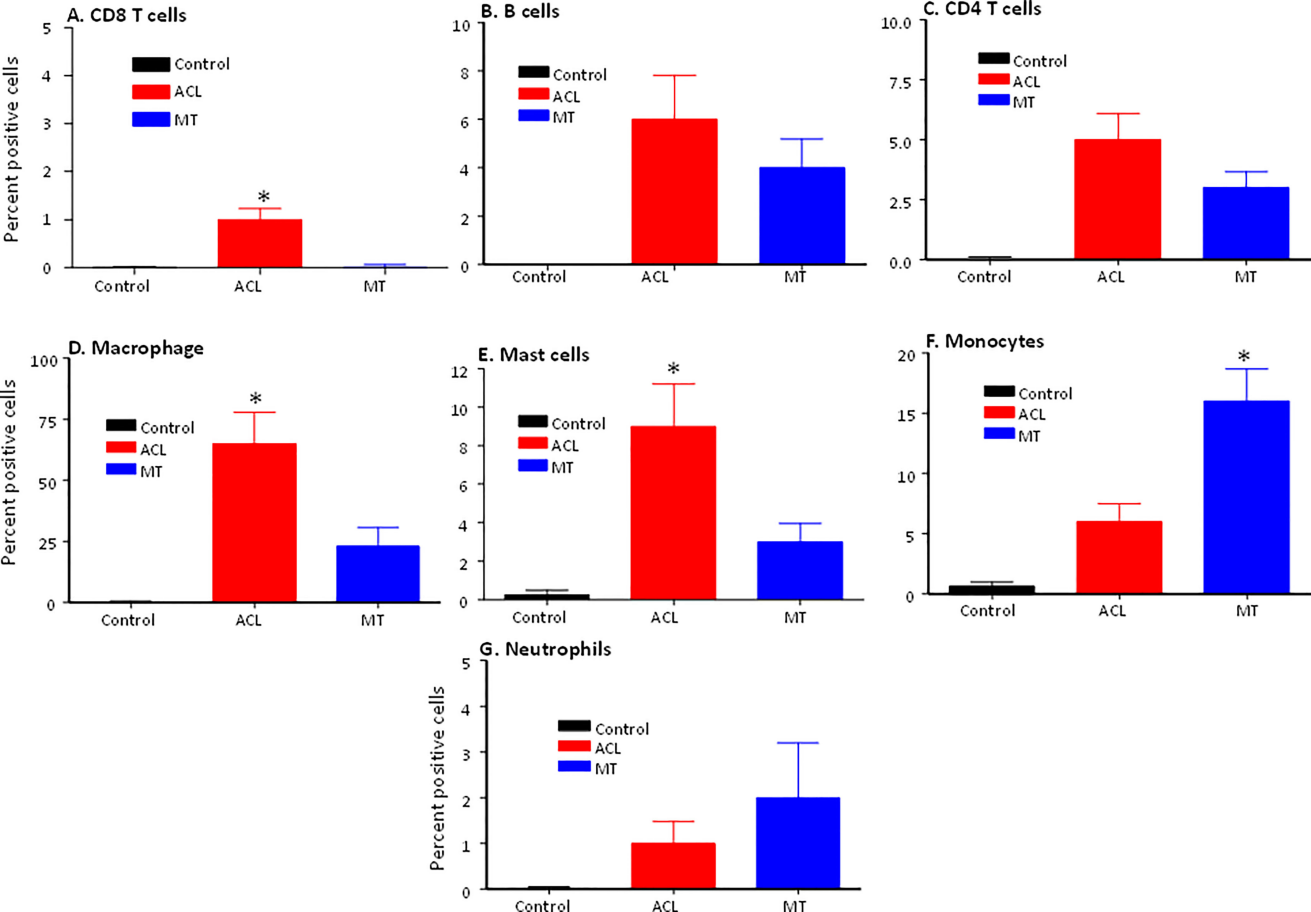
Comparison of percentage of immune and infiltrating cells using MIHC in the DSST after ACL injury and MT at the time of reconstructive surgery and meniscectomy, respectively. **(A)** CD3/CD8+ T cells **(B)** CD19+ B cells **(C)** CD3/CD4+ T cells **(D)** CD68+ Macrophages **(E)** CD117+ Mast cells **(F)** CD14+/CD68- Monocytes **(G)** Neutrophil. Control synovial tissue with no RA no OA n = 4. ACL n = 10 and MT n = 16. Two sections from each DSST were analyzed. Data are shown as Mean ± SEM. *p < 0.05 considered significant.

**Figure 10:**
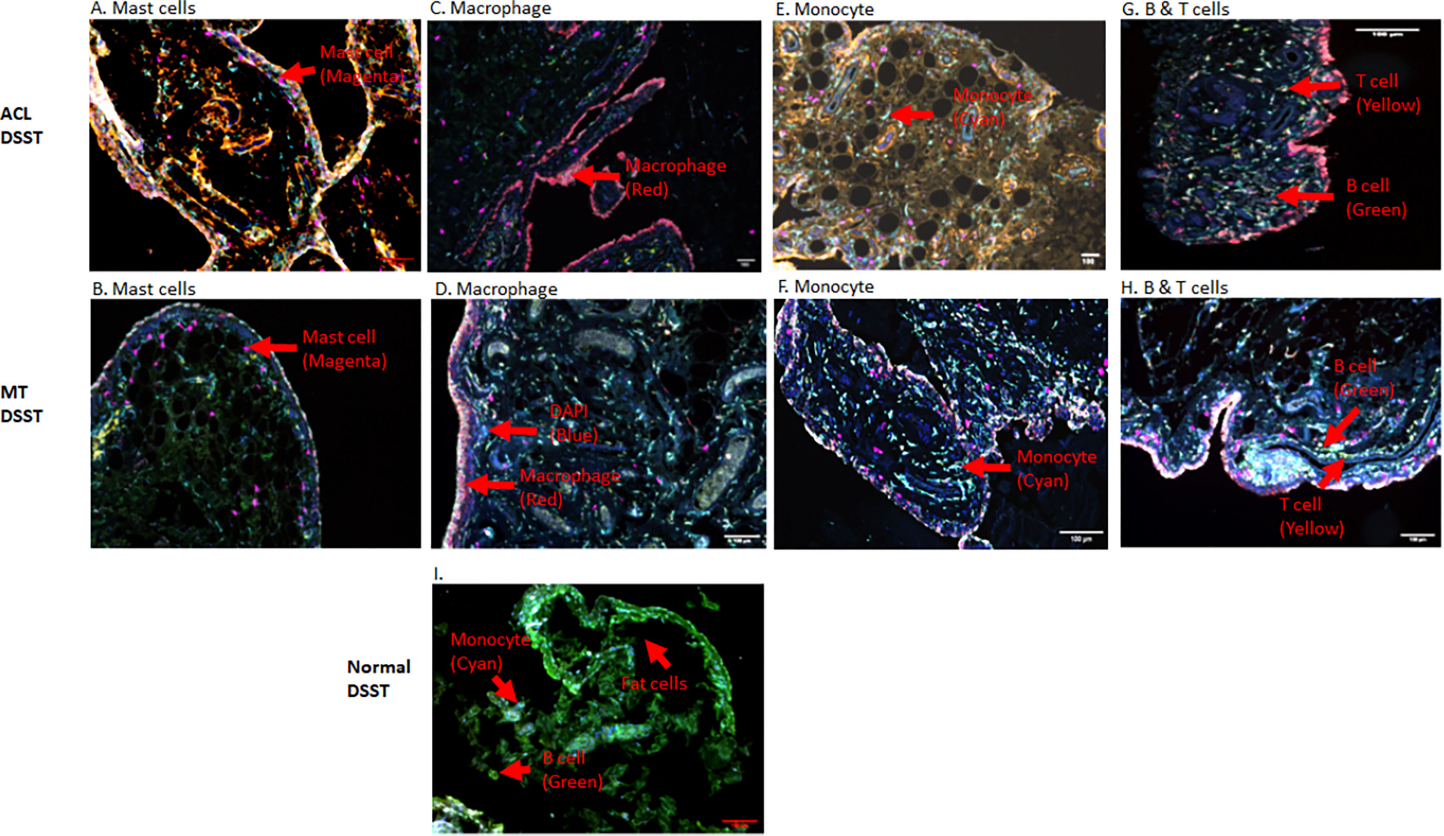
Representative composite images showing the presence of inflammatory cells in the DSST from ACL injury and MT injuries at the time of reconstructive surgery and meniscectomy, respectively. **(A, B)** More mast cells present in ACL synovium (top left panel) compared with MT synovium (second left panel). **(C, D)** More macrophages were present in the ACL synovium (top right second panel) compared with MT synovium (second right second panel). **(E, F)** Less monocytes were present in the ACL synovium (top right third panel) compared with MT synovium (second right third panel). **(G, H)** Few B and T cells were present both in the ACL (top right panel) and MT synovium (second right panel) first. **(I)** Presence of monocytes, fat cells and B cells in normal synovium (bottom panel). Immune and infiltrating cells have been shown using false color coding, including Magenta = Mast cell, Red = Macrophage, Cyan = Monocyte, Yellow = T cell, Green = B cell and DAPI = blue. A red arrow shows the presence of a complement positive cell. A minimum of three composite images were generated using a single DSST with 2 sections from each ACL (n = 10) and MT (n = 16) patients. Normal control DSST (n = 4). Scale bar = 100µm shown at the bottom or top right-hand side or of each composite image.

**Figure 11:**
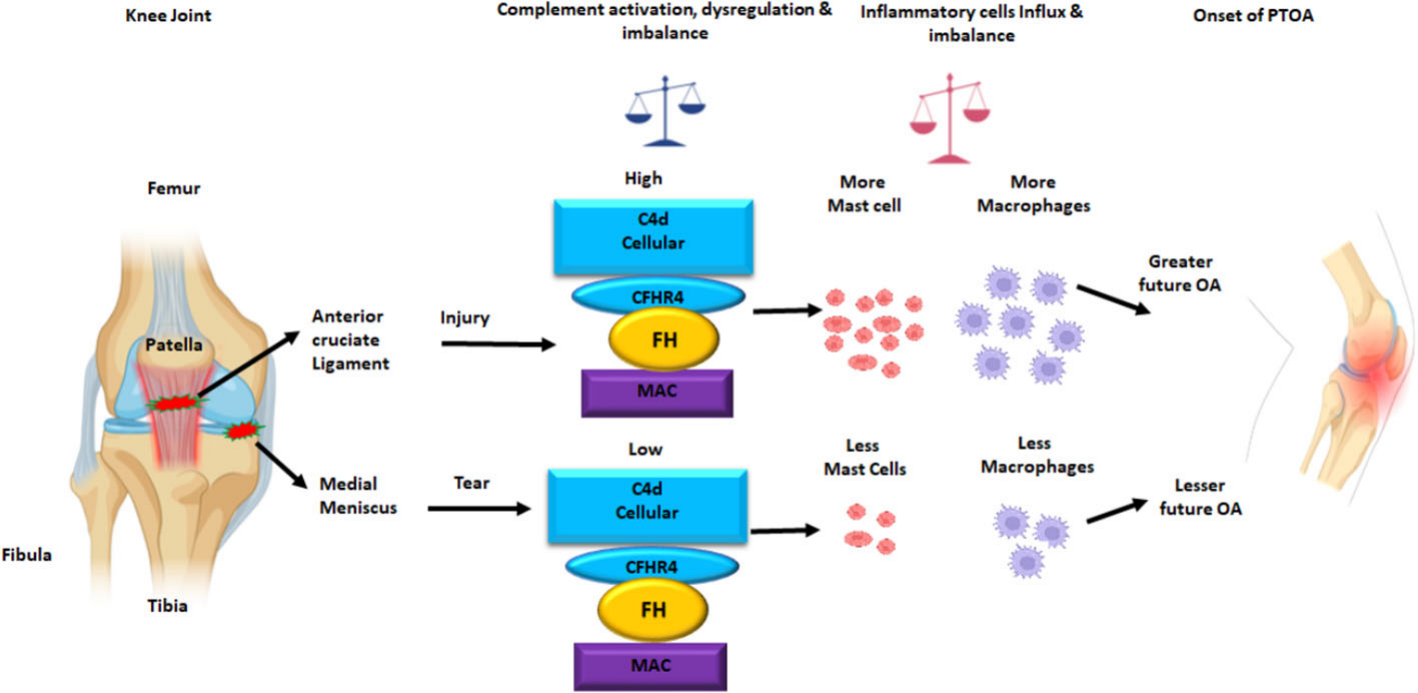
Putative model of the normal knee joint after ACL injury and MT leading an increased C4d, and increased MAC (a.k.a. C5b-9) deposition on the surface of synovial cells in DSST. The CFHR4, a positive regulator of the AP and FH, a negative regulator of the AP protein expression on the surface of synovial cells also increased after ACL injury than MT. There is also an increase and imbalance in the percentage of mast cells and macrophages in the synovium after ACL injury compared with MT. This summary figure shows that differential complement activation, dysregulation and imbalance along with infiltration of innate immune and inflammatory cells might lead to an early or faster onset of OA after ACL than MT injury. All complement proteins, and inflammatory cells have been shown using different colors. This summary figure has been created with Biorender.com.

To determine the presence of specific immune and inflammatory cells, we performed MIHC using DSST on the same samples from the ACL injury and MT patients (**Figure 9**). The percentage of synovial cells expressing CD8 was significantly (p < 0.024) higher in the ACL compared to the MT synovium (**Figure 9A**). In contrast, no significant differences were seen in the percentage of synovial cells expressing CD4 and B cells (**Figures 9B, C**). Notably, the percentages of macrophages (p <0.0025) and mast cells (p < 0.0072) in the ACL synovial tissue were significantly higher than in the MT synovial tissue (**Figures 9D, E**). In contrast, the MT synovial tissue contained a significantly (p < 0.009) higher percentage of monocytes than the ACL synovial tissue (**Figure 9F**). The percentage of monocytes was significantly higher in female MT synovium than female ACL synovium (p < 0.019) (data not shown). Overall, no significant differences were seen in the percentage of neutrophils in the ACL and MT synovial tissue (**Figure 9G**). The percentage of neutrophils in males was significantly (p < 0.04) higher compared with female in the MT DSST synovium (data not shown). Representative MIHC composite images from ACL, and MT DSST show the presence of mast cells (**Figures 10A, B**), macrophages (**Figures 10C, D**), monocytes (**Figures 10E, F**), and B and T cells (**Figures 10G, H**). Normal controls show the presence of few monocytes, abundant fat cells and B cells (**Figure 10I**). The presence of adaptive immune cells in the tissue suggests that adaptive immunity also plays an important role after ACL and MT injuries. These data overall suggest that complement activation after ACL injury leads to the activation of effector cells such as macrophages and mast cells, and that this was more pronounced after ACL injury as shown in a putative model (**Figure 11**). Infiltration of innate and adaptive immune cells into the synovial tissue in ACL and MT was also seen, varying by the type of injury.

## 4 Discussion

In this study, using MIHC, we have compared the presence of several complement proteins, split complement products, complement receptors and complement regulatory proteins, as well as immune and inflammatory cells, in the DSST obtained from OA patients before total knee replacement and arthroscopically after ACL injury, and MT but before reconstructive surgery and meniscectomy, respectively. Notably, synovia from both ACL and MT were substantially infiltrated with cells and showed evidence of marked complement activation. To the latter point, we found that the CP and LP C4 activation fragment C4d was more highly expressed on synovial cells from ACL DSST than the MT DSST, suggesting a higher level of a complement-mediated effect in the synovium after ACL injury as compared to MT. In addition, the AP of the CS was likely to be more dysregulated, due to the higher expression of CFHR4, in the ACL DSST after injury than the MT DSST. Higher levels of C5b-9 (MAC) expressing synovial cells were also present in the ACL DSST compared with MT DSST, again suggesting more complement-mediated damage in the synovium after ACL injury compared to MT. As one means of mediating pro-inflammatory effects, the high expression of C3aR1 and C5aR1 on the surface of synovial cells after ACL injury compared with the MT suggested that local complement-mediated signaling might enhance inflammation after ACL. Beyond the CS itself, the high percentage of mast cells and macrophages in the DSST after ACL injury compared with the MT suggests a more dominant role of the CS after ACL injury than the MT, likely because of the presence of C3aR1 and C5aR1 (37) acting as receptors for pathogenic chemoattractants. After ACL injury, crosstalk between mast cells expressing C3aR1 and C5aR1 and CS is also likely, as C3a and C5a are chemoattractants for mast cells (35). Finally, the major finding of current study is that although both ACL as well as the MT injuries activate the CS in synovium, but ACL injury more significantly dysregulated the AP and injured to the synovium at early stage, which might be one of the contributing factors or potential risk factors for the initiation of inflammatory processes that can lead to the development of OA in the long term.

Additionally, we observed that there were no significant differences in the vascularity, presence of synovial adipocytes, extent of fibrosis and synovial membrane thickness comparing the DSST from ACL and MT injuries (**Figure 2**). However, increased vascularity in the synovial tissue from MT of females compared with the synovial tissue from MT males suggests that there might be more inflammation in females than males after MT. These observations indicate that MT might predispose females to OA disease.

We found a higher percentage of synovial cells expressing C4d specifically in DSST after the ACL injury than in MT (**Figure 4B**). Interestingly C4d is covalently bound to the injured cells (21), and it is widely accepted as one of the markers for antibody-mediated rejection in renal and cardiac allografts (38, 39), but our MIHC shows that it is also present on the synovium after ACL and MT injuries, although differentially. Normally CFI cleaves iC4b into C4d (surface bound) and C4c (soluble) (21). The CS is activated by three pathways: CP, LP and AP, but only the CP and LP share C4; therefore, C4d deposited on the injured or apoptotic cells could be an activation product from CP or LP resulting from binding of natural antibodies (NAbs) to injured tissues. Consistent with our observations, another study has reported the presence of an increased level of C4d in the synovial fluid several years after knee trauma (32); therefore, it could be an informative synovial injury marker after ACL injury.

Healthy individuals express NAbs to protect from viral and bacterial infections, but these can also contribute to autoimmune diseases (40). Furthermore, IgG or IgM NAbs are known to activate the CP or LP. NAbs, which are germline coded as a part of natural autoantibodies (NAAb), also bind to and clear the self-neoepitopes produced by apoptotic and necrotic cells (41). Previous studies have shown that a Nab monoclonal antibody, designated C2, binds to a subset of phospholipids displayed on injured cells in the joints in inflammatory arthritis, as confirmed by the therapeutic benefit on the joint inflammation of C2 Nab-derived scFv-containing protein fused to a complement inhibitor: complement receptor-related y (Crry) (42). Natural IgM binds to C1q to activate complement, and likewise mannan binding lectin (MBL) bound to injured cells can activate through MASPs (43–46). At present we do not know the exact mechanism and whether enhanced C4d deposition in the ACL synovial tissue resulted from CP or from LP activation or which specific pathway is involved, or it is antibody independent or dependent or acting as an opsonin on injured cells. Nonetheless, the presence of an increased percentage of cells expressing C5b-9 (MAC) and C5/C5b in the synovium after ACL injury compared with the MT indicate that more synovial cells are injured due to complement activation, which likely is reflected by more deposition of C4d after ACL injury (**Figure 5F**). In addition, ACL synovium deposition of C4d and C5b-9 is consistent with a previous study in which synovial fluids from OA, RA and knee injury patients were found to contain elevated levels of C4d, C3bBbP, and soluble terminal complement complex (32). Our current C4d deposition data in the ACL synovium are consistent with this study, regardless of the type of knee injury.

In comparison to MT expression of C3aR1 and C5aR1 was increased on the surface of synovial cells after ACL injury, consistent with an important role for these receptors prior to the development of OA. These C3aR1 and C5aR1 receptors data imply that OA development after ACL is more likely than the MT. If so, then our data are consistent with the overall higher risk of developing OA after ACL than the MT after 19 years of follow up (47). C3aR1 and C5aR1 might be involved in local tissue repair similar to their upregulation in multiple sclerosis for demyelination and remyelination (48). Although we have not seen any differences in this cross-sectional study in the percentage of adipocytes in the DSST after ACL injury and MT, the role of obesity has clearly been documented in the development of PTOA, for adipocytes do express C3aR1 and C5aR1 on their surface; in addition, as the BMI in this study patients is in the higher range, we don’t rule out that adipocytes also generate complement factor D (a.k.a. adipsin), especially as lipodystrophic mice do not develop spontaneous or PTOA (49–52). There is likely an association between adipsin, OA synovial membrane and cartilage degradation through activation of the complement system (53). Interestingly, our previous studies have shown that *C3aR1* or *C5aR1* gene targeted mice are resistance to collagen antibody-induced arthritis (54), and there was also a positive correlation between *C5aR1* gene expression and clinical disease activity score 28 in synovium from early RA patients (36), suggesting an important role for C5aR1 in inflammation both in mice and humans. C3aR1 and C5aR1 down-stream signaling results in activation of Mitogen-activated protein kinase (MAPK) and phosphoinositide-3-kinase–protein kinase B/Akt (PI3K-PKB/Akt) pathways (55).

C3aR1 and C5aR1 are normally expressed on neutrophils, basophils, eosinophils, mast cells, monocytes/macrophages and also on adipocytes (50–52, 56). C5aR1 contribute to macrophage accumulation in obese adipose tissue (57). The increased presence of macrophages and mast cells in synovial tissue from ACL injury compared to MT suggests there is a sustained complement-mediated innate immune cellular response after ACL injury. These data also suggest that complement in both injuries might be controlling differentially immune cells mobilization/and or recruitment, as well as inflammation, for there is an increased presence of monocytes in the synovial biopsies obtained after MT (**Figure 9F**). In contrast to the mast cell, macrophage and monocyte phenotypes, no differences were seen in percentage of neutrophils comparing ACL and MT synovial tissue (**Figure 9G**). There were also no differences in the percentage of B and CD4 T cells in the DSST from ACL and MT (**Figures 9B, C**), indicating less involvement of the adaptive immune response in the differential responses after these injuries. The high percentage of mast cells and macrophages in the ACL synovium compared with MT synovium show that these two cell types might trigger a more robust inflammatory response after ACL injury. An increased percentage of synovial mast cells in ACL could be the result of their local proliferation or a chemokine-mediated infiltration at the site of injury. Interestingly other studies have shown that synovial macrophages and lymphocytes are the most predominant immune cells in OA whereas B cells, plasma cells and mast cells are found to a lesser extent (58–60). Thus, after ACL or MT injuries, there might be a change in cellular composition in the long term, but initial effector cells might be different after ACL and MT knee injuries. Consistent with above mentioned studies, our MIHC using separate OA synovial tissue also show that macrophages, mast cells and monocytes are also present (data not shown) which could be responsible for moderate inflammation seen in OA. Mast cells, which also express C3aR1 and C5aR1 were present predominantly in synovial tissue from ACL injury than MT. We speculate that ACL-mediated injury can damage cartilage due to the secretion of tryptase by mast cells which can destroy cartilage after ACL injury and MT. Tryptase has been shown to be released by mast cells through activation of protease-activated receptor 2, which is increased in OA and RA synovium and cartilage (58, 61, 62) however, we have not performed double staining for CD117/Tryptase using ACL and MT DSST. Of note, we noticed that C3aR1 is highly expressed surrounding the blood vessels in the DSST after ACL injury compared with MT (**Figures 8A, B**). C3aR1 are present on vascular endothelial cells (63). Interestingly the role C3a/C3aR1 signaling in diapedesis has also been documented (64) and we don’t rule out the role of C3aR1 in promoting vascular inflammation after these injures. Overall, compared to MT, synovium after ACL injury possesses several key pre-immune features, suggesting that in addition to pathological mechanical cues, ACL injury may initiate development of OA *via* early immune system activation and pro-inflammation.

Furthermore, a high percentage of ACL DSST samples demonstrated features of AP activation, suggesting that release of CFH might be important to control AP activation after ACL injury, with the goal to maintain an anti-inflammatory environment locally for it is a negative regulator of the AP. Thus, CFH might be playing a protective role to control initial synovial inflammation or cell death following ACL injury. Consistent with our study a high expression of both CFH and FHL-1 proteins, in OA synovium, has been also documented previously (65). However, the high expression of both CFHR4 and CFH proteins in the synovium after ACL compared with MT confirm that there is local AP activation in the synovium that may be contributed by CFHR4 dysregulation. Our data show that AP dysregulation in the synovium after ACL injury might be due the presence of an increased percentage of CFHR4+ cells. Therefore, initial synovial damage after ACL injury might in part be contributed to by CFHR4 generation, as CFHR4 counter regulates the AP through competition with CFH. The tight balance between AP activation and regulation due to an elevated expression of CFHR4 in the synovium might be lost after ACL injury compared with the MT. Mechanistically, CFHR4 allows complement activation by binding to C3b and it also binds to C-reactive protein (18). There are two splice variants of *CFHR4* gene i.e., CFHR4A and CFHR4B. We were not able to distinguish between both forms of CFHR4 proein using MIHC due to the lack of specific antibodies, but studies have shown that both act as competitors of CFH (18). In DSST we show that complement proteins of the AP, such as C3c, CFB (**Figures 5A, C**), are expressed locally were not statistically different among ACL, MT and OA.

One of the limitations of the current study is that we have not compared the complement proteins and immune cells in the synovial tissue between different types of ACL tear (partial tear, complete non retracted tear, and complete retracted tear) and MT (radial tear and bucket handle tear) injuries. A second limitation is that we have focused only on the ACL and MT related synovial phenotypes; we have not assessed synovial fluid chemo-attractants such as C3a and C5a, the assessment of which could be additionally informative. Nonetheless, previous studies have shown the presence of higher levels of complement components in the synovial fluid from an early stage of the OA compared with healthy controls (29). We do not rule out the possibility that there were complement activated products or split fragments present before the surgery for surgery is normally done an average thirty days after the injuries and it is rare that reconstructive surgeries and meniscectomies are done immediately on the same day of injury. It is challenging and technically difficult get DSST two times i.e., immediately after the injury and before surgery. Another limitation of current study is that we have not performed sample size justification due to the nature of this study. Additionally, although we have identified some sex-specific differences, we were underpowered in this study to fully characterize sex as a biological variable. We also cannot clearly define which pathway(s) of the complement system initiate complement activation. We found no differences in the percentage of cells expressing FCN3 (data not shown) and MBL2 in the ACL and MT synovial tissue, but we can’t rule out the possibility that other components of these pathways may be involved, including CP (such as C1q) or LP (such as Collectins) or FCN1 and FCN2. LP activation may be involved at late stage of the disease due to high expression of MBL2 in OA vs. ACL and MT, so it is unlikely to be an initial trigger.

In conclusion, the evidence in the current study is consistent with a substantial role for the CS in both types of injury, but with differential mechanisms related to complement and immune cells in the synovium from ACL injury and MT. Further CITE-seq (Cellular Indexing of Transcriptomes and Epitopes by Sequencing) for complement genes-proteins along with specific cell-specific phenotyping antibodies using DSSTs from ACL, MT, OA and uninjured will be highly informative along with the current MIHC data. Overall ACL injury affected the synovium more severely, as evident from an increased C4d deposition, dysregulation of the AP by CFHR4, high expression of C3aR1, C5aR1, C5b-9 and effector cells compared with the MT injury. Our data show that complement-dysregulation in synovium might be playing an important role after these injuries; therefore, complement might be a valid candidate therapeutic target, in the ACL context particularly, with the goal to mitigate or prevent early joint changes that predispose for the onset of PTOA. This study also suggests when, and for what types of injuries we could apply complement therapy to achieve a bon fide clinical benefit. Based on current study, we propose that antagonists of C3aR1 or C5aR1, dual targeting by anti-C5aR1ab-C5siRNA conjugate (66), inhibitors of the LP such MASP2 or inhibitors of the CP and AP such as TT32 and bifunctional fusion protein (67, 68), or inhibitors of the CFHR4 protein or scFv-C4d-CFH could be useful new therapeutic tools to be applied immediately after ACL or MT surgery to prevent or mitigate initiation of OA disease

## Data availability statement

The original contributions presented in the study are included in the article/supplementary material. Further inquiries can be directed to the corresponding author.

## Ethics statement

The studies involving human participants were reviewed and approved by IRB University of Colorado Denver. The patients/participants provided their written informed consent to participate in this study.

## Author contributions

NKB and VMH conceived the idea for analyzing complement proteins and immune cells in the synovium of ACL, MT and OA patients. VMH reviewed this manuscript in-depth. NKB supervised the project, executed the experimental plan, analyzed data and wrote the first, final and revised draft of the manuscript. RMF provided primary and secondary diagnosis, performed all surgical procedures, collected and provided DSST before ACL reconstructive surgery, before MT meniscectomy and also provided her expert advice related to each diagnosis. AC was involved in patient recruitment and routine tissue collection, processing, and preservation of DSST from ACL injury and MT provided by an Orthopedic surgeon at the time of ACL reconstructive surgery or meniscectomy. JS catalogued ACL and MT related DSST samples and kept the patients record securely using RedCap. MZ was involved in the discussions related to this work and reviewing the manuscript. SA, a combined BA/BS-MD program student, generated and analyzed some of the composite images related to the ACL and MT using MIHC. SA also examined ACL and MT H & E-stained histopathology slides and generated data. CS provided DSST from OA patients undergoing a total knee replacement surgery. MRC provided pathology assessment and cross-checked the quality of ACL and MT DSST. LWM identified ACL and MT patients and provided expert advice regarding primary and secondary diagnoses. All authors contributed to the article and approved the submitted version.
